# The phenotypic spectrum associated with *OTX2* mutations in humans

**DOI:** 10.1530/EJE-20-1453

**Published:** 2021-05-05

**Authors:** Louise C Gregory, Peter Gergics, Marilena Nakaguma, Hironori Bando, Giuseppa Patti, Mark J McCabe, Qing Fang, Qianyi Ma, Ayse Bilge Ozel, Jun Z Li, Michele Moreira Poina, Alexander A L Jorge, Anna F Figueredo Benedetti, Antonio M Lerario, Ivo J P Arnhold, Berenice B Mendonca, Mohamad Maghnie, Sally A Camper, Luciani R S Carvalho, Mehul T Dattani

**Affiliations:** 1 Section of Molecular Basis of Rare Disease, Genetics and Genomic Medicine Research & Teaching Department, UCL Great Ormond Street Institute of Child Health, London, UK; 2 Department of Human Genetics, University of Michigan, Ann Arbor, Michigan, USA; 3 Developmental Endocrinology Unit, Hospital das Clinicas da Faculdade de Medicina da Universidade de São Paulo, São Paulo, Brazil; 4 Department of Pediatrics, IRCCS Istituto Giannina Gaslini; 5 Department of Neuroscience, Rehabilitation, Ophthalmology, Genetics, Maternal and Child Health, University of Genova, Genova, Italy

## Abstract

**Objective:**

The transcription factor OTX2is implicated in ocular, craniofacial, and pituitary development.

**Design:**

We aimed to establish the contribution of *OTX2* mutations in congenital hypopituitarism patients with/without eye abnormalities, study functional consequences, and establish *OTX2* expression in the human brain, with a view to investigate the mechanism of action.

**Methods:**

We screened patients from the UK (*n* = 103), international centres (*n* = 24), and Brazil (*n* = 282); 145 were within the septo-optic dysplasia spectrum, and 264 had no eye phenotype. Transactivation ability of *OTX2* variants was analysed in murine hypothalamic GT1-7 neurons. *In situ* hybridization was performed on human embryonic brain sections. Genetically engineered mice were generated with a series of C-terminal *OTX2* variants.

**Results:**

Two chromosomal deletions and six haploinsufficient mutations were identified in individuals with eye abnormalities; an affected relative of one patient harboured the same mutation without an ocular phenotype. OTX2 truncations led to significant transactivation reduction. A missense variant was identified in another patient without eye abnormalities; however, studies revealed it was most likely not causative. In the mouse, truncations proximal to aa219 caused anophthalmia, while distal truncations and the missense variant were tolerated. During human embryogenesis, *OTX2* was expressed in the posterior pituitary, retina, ear, thalamus, choroid plexus, and partially in the hypothalamus, but not in the anterior pituitary.

**Conclusions:**

*OTX2* mutations are rarely associated with hypopituitarism in isolation without eye abnormalities, and may be variably penetrant, even within the same pedigree. Our data suggest that the endocrine phenotypes in patients with *OTX2* mutations are of hypothalamic origin.

## Introduction

The transcription factor orthodenticle protein homologue 2 (Otx2/OTX2) plays a distinct role in the patterning of the anterior neuroectoderm, midbrain, forebrain, pituitary gland and sensory organs including the inner ear, the pineal gland, and the eye (namely the optic nerves and retinal photoreceptors) (1, 2, 3, 4). It is also important for the regulation of other transcription factors, such as *Hesx1*, which is essential for forebrain, pituitary and eye development (5, 6). Loss of murine *Otx2* function due to a homozygous genetic mutation is associated with embryonic lethality due to complete loss of the forebrain, whilst heterozygous *Otx2* mutant mice manifest a variable phenotype ranging from no obvious craniofacial anomaly to having microphthalmia/anophthalmia, otocephaly/micrognathia, and even acephaly in extreme cases (7, 8, 9). Conditional knockout of *Otx2* in the retina leads to a failure in photoreceptor development (10), while conditional knockout of *Otx2* with *Foxg1-cre* in limited brain regions and the oral ectoderm (the prospective anterior pituitary) causes craniofacial defects with abnormal pituitary gland morphology. Conditional knockout in the neural ectoderm (prospective posterior pituitary) results in a hypoplastic anterior lobe, posterior lobe and pituitary stalk, with loss of FGF signalling, which impairs anterior lobe growth. Neither of the latter two mutants, however, affect differentiation into the five specialized anterior pituitary cell types (3). Furthermore, mice elicit a hypogonadotropic hypogonadism phenotype when *Otx2* is conditionally knocked out in GnRH neurons (11).

In humans, mutations in *OTX2* have been described in patients with eye defects and variable congenital hypopituitarism (CH), ranging from isolated growth hormone deficiency (IGHD) (12) to combined pituitary hormone deficiency (CPHD) with/without an ectopic posterior pituitary (EPP) (6, 13, 14, 15, 16, 17). Heterozygous mutations, including chromosomal deletions that span the *OTX2* gene, have been implicated in major developmental malformations related to the eye. These include microphthalmia and anophthalmia in 2–3% of cases (14, 18), retinal dystrophies such as Leber congenital amaurosis (LCA) and pigmentary retinopathy, coloboma, and optic nerve hypoplasia (ONH) (4, 13, 14). The frequencies of eye abnormalities and pituitary insufficiency in patients with *OTX2* variants are 78 and 30%, respectively (15). Only two *OTX2* variants have been described in association with pituitary dysfunction without an ocular phenotype; p.N233S and p. R127W (11, 19, 20).

A recent summary of all previously published *OTX2* mutations reported that sporadic, *de novo*, familial dominant with complete penetrance, and familial dominant with incomplete penetrance mutations accounted for 37%, 42%, 16%, and 5% of cases, respectively (21). The frequency of co-existence of eye and pituitary defects remain unclear. However, because there could be ascertainment bias in screening and because visual impairment is of greater concern and may present with a more obvious phenotype than endocrine deficiency, the endocrine function may not be as thoroughly evaluated in patients with eye malformations.

We sought to establish the frequency of *OTX2* variants in our large multi-ethnic cohort of patients with hypopituitarism in the presence and absence of eye abnormalities and to study their functional consequence. Using *in situ* hybridization, we also aimed to clarify a detailed human embryonic brain *OTX2* expression profile, with a particular focus on the hypothalamic-pituitary region, in order to better understand the mechanisms by which *OTX2* mutations lead to variable clinical phenotypes.

## Subjects and methods

### Patients

#### The UK cohort

A total of 127 patients with a CH diagnosis within the spectrum of septo-optic dysplasia (SOD) were recruited between 1998 and 2019; 103 from UK national centres and 24 were recruited internationally. Ethical committee approval was obtained from the UCL Great Ormond Street Hospital Institute of Child Health/Great Ormond Street Hospital for Children Joint Research Ethics Committee, and informed written consent was obtained from patients and/or parents. MRI was available for 60% (*n* = 75) of the patients, of which 46% (*n* = 35) had an EPP. Eye phenotypes ranged from bilateral microphthalmia/anophthalmia (*n* = 9), unilateral microphthalmia or anophthalmia (*n* = 12), to coloboma (*n* = 2), cataract (*n* = 1) and retinal dysplasia (*n* = 3), with the remaining having ONH as their only eye phenotype (*n* = 100). All patients had previously been screened and tested negative for mutations in *HESX1*, *SOX2*, *SOX3*, and *PROKR2.*

#### The Brazilian cohort

A total of 282 patients were studied; 82 with IGHD (4 with SOD) and 200 with CPHD (12 with SOD) with and without eye abnormalities. All 282 patients had an MRI performed, with 28 IGHD patients and 119 CPHD patients having an EPP. We obtained informed consent from the parents or patients to study their DNA and to include clinical photographs in this manuscript where relevant. The protocol was approved by the Hospital das Clinicas Ethics Committee. These patient DNA samples were sequenced at the University of São Paulo and the University of Michigan. The latter received anonymized samples that were determined to be exempt from IRB approval.

### Mutation analysis

#### UK cohort

The coding region of human *OTX2* (NM_172337) was amplified by the PCR (conditions available upon request). PCR products were treated with MicroClean reagent (Web Scientific, Cheshire, UK), and exons and intron–exon borders were sequenced using BigDye version 1.1 sequencing chemistry (Applied Biosystems) and analysed on a 3730X1 DNA analyser (Applied Biosystems).

#### Brazilian cohort

Patient DNA samples were screened by PCR for *OTX2* coding exons and intron–exon borders (11), by an established gene panel (22), or by exome sequencing (21 cases) (23).

### Functional studies

#### Expression constructs and site-directed mutagenesis

The single nucleotide variants, deletion and insertion/deletion that were detected in the patients were introduced into the pCT expression vector containing the full-length coding region of WT human *OTX2*, using the QuikChange II XL Site-Directed Mutagenesis Kit (Agilent). DH5α cells were transformed with each mutant *OTX2* construct, respectively, and the sequence was verified by the Advanced Genomics Core at the University of Michigan.

#### Cell culture and transfection for quantitative analysis of transactivation

GT1-7 cells (provided by Pamela Mellon, University of California, San Diego (24)) were maintained in a humidified CO_2_ (5%) incubator at 37°C in Dulbecco’s minimum essential medium (DMEM) supplemented with 10% foetal bovine serum (FBS, Gibco), 5% penicillin–streptomycin. GT1-7 cells were seeded at 150 000 cells/well in 24-well plates before transient co-transfection 24 h later with 8 ng/well of wt/mutant pCT-h*OTX2* expression constructs, 256 ng/well luciferase reporter containing *OTX2* consensus binding sites, and 16 ng/well renilla reporter constructs, made up to a total of 400 ng/well with empty pcDNA3.1 vector (also containing the CMV promoter), which served as the control. Fugene-6 (Promega) transfecting agent was used at a 3:1 ratio to total DNA. Cells were incubated for 24 h at 37°C prior to lysis and processed for luciferase activity using the Dual-Luciferase Reporter Assay system (Promega), and read on the FluorStar Optima (BMG technologies). Three independent experiments in triplicate were carried out. Values were normalized to Renilla luciferase and results then normalized to empty vector.

#### Mouse studies

All procedures were conducted in accordance with the principles and procedures outlined in the National Institutes of Health Guidelines on the Care and Use of Experimental Animals and approved by our Institutional Animal Care and Use Committee (PRO00008702). Mice were housed in an AALAC approved animal facility at the University of Michiganin a 12 h light:12 h darkness cycle in ventilated crates with unlimited access to tap water and Purina 5020 chow.

The *Otx2* variant alleles were generated by microinjecting enhanced specificity Cas9 protein (30 ng/uL, Integrated DNA Technologies), DNA donor oligo (10 ng/µL, Integrated DNA Technologies), and C77G2 crRNA 10 ng/μL annealed with tracrRNA 15 ng/μL (Integrated DNA Technologies) into fertilized eggs obtained from super-ovulated B6CBAF1 females purchased from the Jackson Laboratory. Pronuclear microinjection was performed as described (25). Founders with alterations were bred to CBA or B6 to produce progeny. Some progeny were intercrossed to produce homozygotes for the mutations.

For the genotyping of *Otx2^H230L^* and Otx2^L219fs*17^ alleles, a 582 bp fragment was amplified by PCR (details are available upon request). The wt PCR product was 582 bp, while the restriction enzyme digestion (HpaII or MspI) of the H230L allele resulted in 301 and 281 bp fragments. The L219fs*17 allele was analysed by Sanger sequencing using the same primers used in the PCR.

#### Histological analyses

For analysis of murine neonatal pituitary glands, P0 heads were fixed with 4% paraformaldehyde (PFA) overnight following the dissection and removal of the skin, lower jaw, and skull. After fixation, the heads were treated with 10% EDTA overnight to demineralize the bone. Adult eyes were fixed with 4% PFA. Heads and adult eyes were dehydrated in graded ethanol, embedded in paraffin, and sectioned to a 5 µm thickness and mounted onto microscope slides before analysis.

#### Immunostaining

Deparaffinization and hydration of paraffin-embedded samples were performed with xylene and graded alcohol and 1× PBS. Primary antibodies against GH (monkey anti-GH, 1:100 dilution) and LHb (guinea pig anti-LH, 1:100 dilution) were purchased from the National Hormone and Peptide Programme. Biotinylated anti-human biotin and anti-guinea pig were used as the secondary antibodies. Strep-FITC was used for detection. Images of HE staining and immunostaining were obtained with a DFC7000 T (Leica Microsystems). Images were processed using LasX software (Leica Microsystems).

#### In situ hybridisation on human tissue sections

A purified pCMV-SPORT6 vector containing full-length human wt*OTX2* cDNA (IMAGE ID:5493541) (Source Bioscience) was used to make both the antisense and control sense digoxigenin-labelled RNA probes. Human embryonic tissue sections were selected at Carnegie stage (CS) 19, 20 and 23 (equivalent to gestational age (GA) 6, 7 and 8 weeks into development), respectively, obtained from the Human Developmental Biology Resource (HDBR). The *in situ* hybridization protocol was carried out as previously described (26), to generate a human embryonic expression brain profile incorporating the hypothalamic-pituitary region. Conditions and details of restriction enzymes and RNA sequences are available upon request.

## Results

### Mutation analysis

#### UK cohort

**Figure 1:**
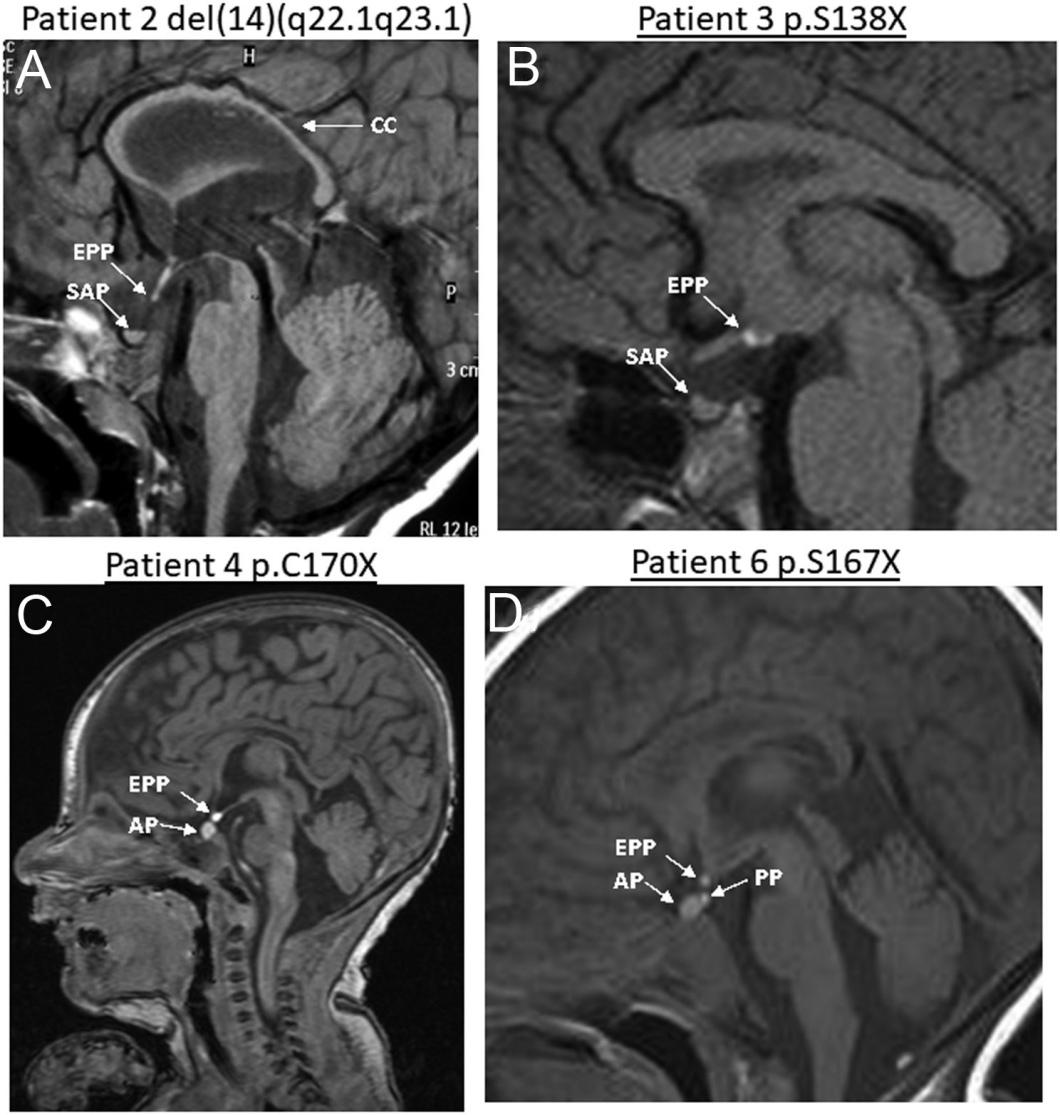
Representative MRI from patients 2–6 illustrates the spectrum of phenotypes in patients with *OTX2* mutations. (A) The MRI image from patient 2 shows a thin corpus callosum (CC), a small anterior pituitary (SAP), and an ectopic posterior pituitary (EPP). (B) The image from patient 3 shows a SAP and an EPP. (C and D) Images from patients 4 and 6 show a normal anterior pituitary (AP) and an EPP, with patient 6 also having a posterior pituitary (PP) bright spot in the sella.

Genetic analysis of 127 patients with SOD and hypopituitarism revealed 7 (5.5%) patients with *de novo* heterozygous mutations in *OTX2* (Fig. 1), including two chromosomal deletions: del(14)(q22.2–23.31) and del(14)(q22.1–q23.1), respectively, a previously reported nonsense mutation c.413C>G, p.S138*, a variant previously identified in a genome-wide study (details in Discussion section) c.510C>A, p.C170*, and 3 novel variants; c.235G>T, p.E79*, c.500_512del13insA (delCCTCTTCCTGCAT), p.S167* resulting from a 13 bp deletion and a single alanine bp insertion, and c.416_420delTCCCG, p.Val139Asp*39 resulting from a 5 bp deletion. These variants are not present in control databases, including the gnomAD Browser (https://gnomad.broadinstitute.org).

#### Brazilian cohort

**Figure 2:**
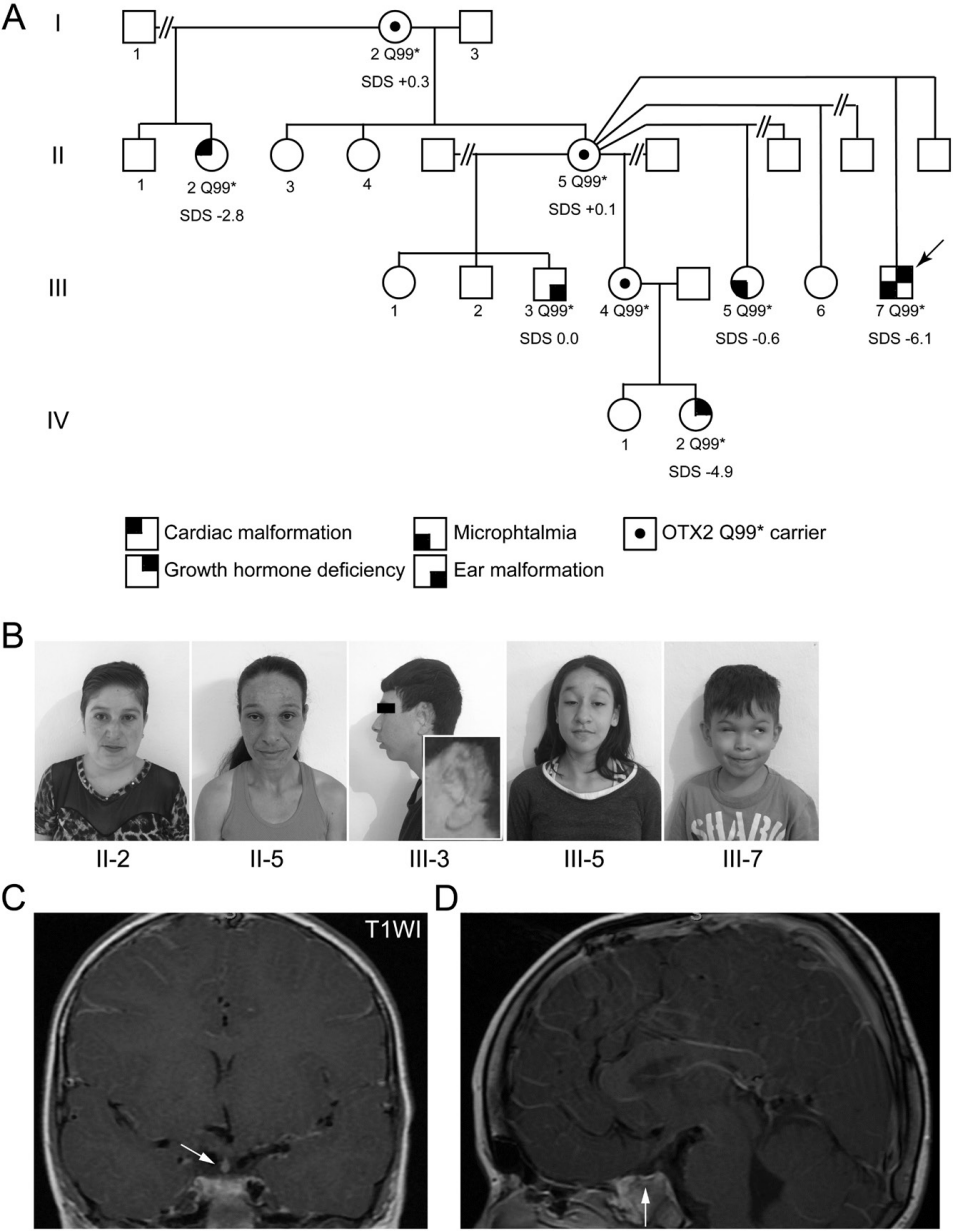
Characteristics of the pedigree with the OTX2 p.Q99* mutation. (A) Pedigree of patients 8 and 9. Incomplete penetrance and variable expressivity are presented through the different annotated phenotypes in affected family members that carry the p.Q99* mutation. (B) Photographs of the proband and other family members. (C and D) MRI of the pituitary showing pituitary stalk interruption. (C) Coronal view: the arrow indicates an ectopic posterior lobe. (D) Sagittal section: arrow indicates a shallow sella with anterior pituitary hypoplasia. Patient III.7 has been previously published (22).

Genetic analysis of 282 patients with congenital hypopituitarism with and without eye defects revealed two heterozygous *OTX2* variants: the previously reported c.295C>T, p.Q99* (13, 22) in an extended family with variable phenotypes (Fig. 2), and a rare variant of unknown significance, c.689A>T, p.H230L, that our studies have now shown to be most likely not causative of the patient phenotype (Supplementary Fig. 1, see section on supplementary materials given at the end of this article). The histidine in the latter substitution is conserved across vertebrate species and *in silico* analyses predict that the leucine substitution is deleterious; therefore, this variant was investigated for functional consequence. At the start of this project, the p.H230L variant was absent from control databases, however recently two heterozygotes were reported in the gnomAD Browser.

### Characteristics of the patients

Patients 1–7 are from the UK cohort, and patients 8–10 are from the Brazilian cohort.

#### Patient 1 (del(14)(q22.2–23.31))

This Caucasian male patient was born at 36 weeks GA with a weight of 2.1 kg (−1.62 SDS). He had bilateral anophthalmia, low set ears, and dysmorphic features. His MRI revealed marked plagiocephaly and generalized white matter loss with a thin corpus callosum. The anterior pituitary was hypoplastic and the posterior pituitary was not visualized. He had severe developmental delay, hearing loss, scoliosis, cryptorchidism, hypoventilation with chronic respiratory failure, and a history of seizures, with no associated hypoglycaemic episodes (Table 1). GHD (peak GH 2.8 µg/L in response to glucagon) was diagnosed at the age of 3.6 years (Table 2). He was treated with recombinant human GH (rhGH, 0.026 mg/kg/day) until the age of 10.5 years when he developed a life-threatening respiratory illness necessitating intensive care. Although he grew well initially with a first-year growth velocity of 10.6 cm/year, the overall long-term response to GH treatment was modest. He commenced in spontaneous puberty at the age of 11.5 years and died at the age of 14 years following a respiratory illness.

**Table 1:** Patients with pathogenic OTX2 variants.

Patient	M/F	Heterozygous mutation	OTX2 protein (NM_172337)	Phenotype	Eyes	MRI
1	M	Gene deletion	del(14) (q22.2–23.31)	GHD, severe developmental delay, hearing loss, scoliosis, cryptorchidism.	BL anophthalmia	Absent PP, small AP, thin CC, plagiocephaly, WML.
2	M	Gene deletion	del(14) (q22.1-q23.1)	GHD, central hypothyroidism, HH, autism spectrum disorder, severe cognitive delay, neural tube defect, cryptorchidism, L1 vertebral schisis, a hypoplastic right kidney.	Unilateral right anophthalmia, coloboma, unilateral left microphthalmia, ONH.	EPP, small AP, thin CC.
3	M	c.413 C>G	p.S138*	GHD, developmental delay, dysmorphic features.	Retinal dystrophy	EPP, small AP, small infundibulum, fusion of anterior fornices, hypothalamic hamartoma.
4	M	c.510C>A	p.C170*	Growth failure	Retinal dystrophy, intermittent nystagmus and a mild maculopathy with vascular flecks.	EPP, normal AP.
5	F	c.235G>T	p.E79*	GHD, cognitive delay, central hypothyroidism	BL microphthalmia, ONH with absence of the optic chiasm and optic tracts, bilateral partial aniridia and right eye coloboma.	EPP, small AP.
6	M	c.500_512del13insA (delCCTCTTCCTGCAT)	p.S167*	GHD, intellectual disability.	Severe BL microphthalmia, optic chiasm aplasia with ONH.	EPP, duplicated PP, normal AP.
7	F	c.416_420 delTCCCG	p.V139D*39	Microcephaly, conductive hearing loss with BL microtia and meatal atresia, a small narrow palate.	BL microphthalmia, ONH.	EPP, small AP, malformation of brainstem, thin CC, incomplete rotation of hippocampi
8	M	c.295C>T	p.Q99*	GHD, delayed neuropsychomotor development.	Right eye microphthalmia, BL nystagmus, right optic nerve atrophy.	EPP, small AP.
9	F	c.295C>T	p.Q99*	GHD	Normal optic nerves, no abnormalities.	EPP, small AP, pituitary stalk present but patent.

Please note that Patient 8 has been previously published in Endocrine Connections (22), 2019 **8** 590–595.

AP, anterior pituitary; BL, bilateral; CC, corpus callosum; EPP, ectopic posterior pituitary; GH, growth hormone; GHD, growth hormone deficiency; HH, hypogonadotropic hypogonadism; M/F, male/female; ONH, optic nerve hypoplasia; PP, posterior pituitary; TSH, thyroid stimulating hormone; WML, white matter loss.

**Table 2:** Endocrine investigations of patients 1–10.

	Patients									
	1	2	3	4	5	6	7	8	9	10
Variant	del(14) (q22.2–23.31)	del(14) (q22.1-q23.1)	p.S138*	p.C170*	p.E79*	p.S167*	p.V139D*39	p.Q99*	p.Q99*	p.H230L^§^
Age at testing, years	3.3	4	10.5	0.6	4	1.3	6.1	7.0	4.5	15
Height SDS	−2.73	−3.6	−2.47	−2.1	−2.1	−0.6	−1.7	−6.1	−4.9	−4.8
BMI SDS	−1	−1.5	−2.96	−3.15	−1.9	−5.4	−2.38	−0.52	+0.79	−1.91
GH peak, µg/L	2.8	4.88, 5.44^†^	10.0	NA	3.52, 5^†^	2.4	28.7	0.7	NA	0.3
Cortisol peak, nmol/L	596	800	947	599	689	880	648	245.58^□,†□^	NA	132.4^□,‡^
FT4, pmol/L	22.7	8.8	12.3	14.4	10.4	12.3	15.2	15.0	NA	7.72
NR	10–22	8–17	10–22	9–19.6	8–17	9–19.6	10.8–19	12–25		7.72–19.8
TSH, mU/L	3	0.8	1.4	4.9	1.05	1.4	1.6	5.96	NA	2.04
NR	<6	0.4–4	0.53–5.27	<6	0.4–4	<6	<6	0.27–4.2		0.5–4.9
IGF1, ng/mL	24	<25	61	<25	<25	<25	35	<20	<20	15
NR	20–170	34–258	111–551	57–327	34–258	51–303	55–248	83.6–361.6	83.6–361.6	335–802
IGFBP3, mg/L	1.45	1.94	2.27	1.38	2.3	1.67	1.59	<0.8	NA	1.3
NR	1.2–4.1	1.8–4.9	2.4–8.4	0.8–3.6	2.2–4.8	0.8–3.6	1.4–6.1	1.8–7.0		1.5–10.9

NR for GH peak: >6.7 µg/L; NR for cortisol peak: >550 nmol/L.

^§^Tolerated variant; ^□^Basal values; ^†□^NR: 184.8–623.5 nmol/L; ^‡^NR: 193.1–855.3.

NA not available; NR, normal range; SDS; standard deviation score †Two types of GH test were performed for the patient (explained in the manuscript).

#### Patient 2 (del(14)(q22.1q23.1))

This male Italian patient was born at 39 weeks GA with a normal weight of 2.8 kg (−1.65 SDS). Villocentesis revealed 46 XY karyotype. Elective caesarean section was indicated for breech presentation; APGAR score was 6–9. Right anophthalmia, left microphthalmia, hypoplastic scrotum, bilateral cryptorchidism and micropenis were noted. MRI revealed a thin corpus callosum and optic chiasm hypoplasia, a small anterior pituitary, and an EPP (Fig. 1A). GHD was diagnosed at the age of 4 years (Table 2) (peak GH of 5.44 µg/L after an arginine test; peak GH of 4.88 µg/L after a glucagon test), and GH treatment commenced; height velocity after the first year of treatment with rhGH at the dose of 0.018 mg//kg/day was 12.5 cm/year (SDS +3.03). He showed early signs of puberty (testes volumes: 4 mL right, 5 mL left) at 13.6 years, followed by a lack of progression. Gonadotrophin response to GnRH test and baseline testosterone were compatible with the diagnosis of central hypogonadism and treatment with testosterone was started at the age of 15 years. His last evaluation was at 15.5 years of age (weight 27.9 kg, height 138.1 cm (−5.0 SDS), and delayed bone age of 1.5 years). His clinical phenotype was characterized by severe cognitive delay, autistic spectrum disorder, central hypothyroidism, central hypogonadism, neural tube defect, L1 vertebral schisis, a hypoplastic right kidney, and cryptorchidism for which he had an orchidopexy at 6 years of age (Table 1).

#### Patient 3 (p.S138*)

This Caucasian male patient was born at term with a weight of 4 kg (+0.95 SDS) and no postnatal problems. From the age of 13 months he was noted to have developmental delay and some dysmorphic features. Ocular evaluation at that stage revealed retinal dystrophy. MRI revealed a small anterior pituitary with a small infundibulum, an EPP (Fig. 1B) with a possible hamartoma in the floor of the third ventricle, and fusion of the anterior fornices (Table 1). A glucagon test performed at the age of 10 years revealed a normal GH peak of 10 µg/L with a normal cortisol concentration; however, rhGH treatment (0.022 mg/kg/day) was started at the age of 12 years, due to growth failure with a low IGF1 concentration (61 ng/mL), suggestive of IGF1 deficiency (Table 2). The family subsequently moved abroad, and he was lost to follow-up.

#### Patient 4 (p.C170*)

This Caucasian boy was born at 40 weeks GA with a weight of 3.2 kg (−0.86 SDS). Clinical evaluation performed at birth revealed absent bilateral red reflexes. MRI of the brain and pituitary revealed cerebellar vermis rotation with a normal-sized vermis, a normal anterior pituitary and an EPP (Fig. 1C). An ophthalmology review performed at the age of 3 months revealed intermittent nystagmus and mild maculopathy with vascular flecks (Table 1). Electroretinography showed absent rod-cone function, suggestive of rod-cone retinal dystrophy. Endocrine evaluation at the age of 3 months showed an undetectable IGF1 concentration (<25 ng/mL). He developed an early growth failure and was too young for a glucagon stimulation test (Table 2). Given the presence of a structurally abnormal pituitary gland and a poor growth velocity with a low IGF1, he was commenced on GH treatment (0.031 mg/kg/day) at the age of 7 months (height −2.1 SDS). The first-year growth velocity on GH treatment was 16 cm/year. He had severe feeding difficulties and was tube-fed via a percutaneous enterostomy. At the last evaluation at 7.7 years of age, his height was 122.1 cm (−0.46 SDS) and weight was 20.2 kg (−1.6 SDS), and he has, therefore, responded well to GH treatment.

#### Patient 5 (p.E79*)

This female Italian patient was born at 36 weeks GA with a weight of 2.9 kg (+0.45 SDS). Clinical evaluation at birth revealed severe bilateral microphthalmia. MRI confirmed anterior pituitary hypoplasia (APH), an EPP, and ONH with an absence of the optic chiasm and optic tracts. She also had bilateral partial aniridia and right eye coloboma. She had both growth failure and cognitive delay. A diagnosis of GHD was made at 4 years of age (Tables 1 and 2) (GH peak of 5.0 µg/L following an arginine test; GH peak of 3.52 µg/L following an insulin tolerance test (ITT)), with normal basal cortisol of 303 nmol/L and a peak of 689 nmol/L after ITT. GH therapy was commenced and height velocity after the first year of treatment with rhGH, at a dose of 0.016 mg//kg/day, was 10.5 cm/year (SDS +2.6), with subsequent development of central hypothyroidism. At the last evaluation at the age of 18 years, her height was 161.9 cm (−0.1 SDS), with a weight of 43.9 kg and a BMI of 16.7 (−1.77 SDS).

#### Patient 6 (p.S167*)

A 7 year old Caucasian boy was born at 41 weeks GA following *in vitro* fertilization (IVF) with a normal weight of 3.36 kg (−0.48 SDS). Clinical examination at birth showed severe bilateral microphthalmia. He had intellectual disability and growth failure (11.3 cm/year in first year of life), with GHD diagnosed at the age of 1.4 years (GH peak on glucagon test of 2.4 µg/L) when GH treatment was started (0.019 mg/kg/day). His hearing was normal. MRI showed a normal anterior pituitary and a duplicated posterior pituitary signal, with bright signals noted in the sella and ectopically in the infundibular recess (Fig. 1D). He also has optic chiasm aplasia with ONH (Table 1). He was commenced on GH with an excellent response; the first-year growth velocity on GH treatment was 8.1 cm/year. At the last evaluation at the age of 8.5 years, his height was 130.8 cm (+0.3 SDS) with a weight of 22kg (−1.5 SDS) and BMI of 12.8 (−2.8 SDS). The rest of his anterior pituitary function remains normal (Table 2).

#### Patient 7 (p.Val139Asp*39)

This female patient was born at term with a weight of 4.0 kg (+1.23 SDS). Antenatal scans were normal but immediately after birth, it was noted that she had poorly formed eyes and ears, and she was subsequently referred for tertiary care. There was no significant history of jaundice or hypoglycaemia. Her clinical phenotype included severe bilateral microphthalmia, bilateral microtia and meatal ear atresia with maximum conductive hearing loss, microcephaly, and a small narrow palate. She is the only child of non-consanguineous Filipino parents, and there is no family history of growth problems. Her height had always been tracked along the second centile (her mid-parental height is just above the 50th percentile). A glucagon test performed at the age of 6 years revealed a normal GH peak of 28.7 µg/L with low concentrations of IGF1 (35 ng/mL) and IGFBP3 (1.59 mg/L) (Table 2). Her height was 105.3 cm (−2 SDS), weight 13.95 kg (−3.2 SDS), and BMI 12.58 (−2.6 SDS). MRI performed at the age of 6 years confirmed bilateral microphthalmia, which was worse on the right side. The optic nerves and chiasm were hypoplastic. The anterior pituitary gland was hypoplastic, with an EPP along the tuber cinereum. A malformation of the brainstem characterized by the midbrain being disproportionately smaller than the pons, a dysplastic corpus callosum and quadrigeminal cistern, and an incomplete rotation of the hippocampi were also noted on MRI (Table 1). At the last review, at 6.6 years of age, her height was 111.8 cm (−1 SDS) with a weight of 15.85 kg (−2.44 SDS), and a height velocity of 12 cm/year.

#### Patient 8 (p.Q99*)

A male patient was born at term with a weight of 3.45 kg (+0.11 SDS) and measured 47 cm (−1.5 SDS). He had delayed neuropsychomotor development, right-sided microphthalmia and bilateral nystagmus (Table 1). At 7 years of age, he presented with severe short stature with a height of 87 cm (−6.1 SDS) and a bone age of 2 years (Table 2). He had a GH peak of 0.7 µg/L following an ITT with an undetectable IGF1 (Table 2). He was commenced on rhGH at a dose of 0.033 mg/kg/day and had a growth velocity of 7 cm/year. Thyroxine treatment was commenced in view of a low normal FT4 in conjunction with a slightly elevated TSH (free T4 1.17–1.27 ng/dL (NR 0.93–1.70) and TSH 5.56–5.96 µIU/mL (NR 0.27–4.20)). His MRI showed an EPP, APH (Fig. 2C and D), and right optic nerve atrophy (data not shown). The index case (III.7 in Fig. 2A) has been previously reported (22), with a heterozygous *OTX2* c.295C>T, p.Q99* variant identified on a targeted gene panel and inherited from his asymptomatic mother (II.5) and grandmother (I.2) (height +0.1 SDS and +0.3 SDS, respectively) (Fig. 2A). The variant was also detected in his half-sister (III.5) with microphthalmia, nystagmus and a normal height (height −0.6 SDS), in his half-brother (III.3) with divergent strabismus (height −0.03 SDS), ear malformations and hypoplasia of the external auditory canal (Fig. 2A and B), and interestingly in another unaffected half-sister (III.4). One maternal aunt (II.2) harbouring the variant, without facial abnormalities, was born with tetralogy of Fallot. She also had short stature (height −2.8 SDS) and a low IGF1 (Fig. 2A and B). Another unaffected half-sister (III.6) was sequenced and did not harbour the variant (Fig. 2A).

#### Patient 9 (p.Q99*)

The niece (IV.2) of patient 8 (III.7), whose unaffected mother (III.4) carries the variant, was born at 40 weeks GA with a birth weight of 3.08 kg (−0.3 SDS) and measured 41 cm (−4.3 SDS). She had jaundice requiring phototherapy for 4 days. At 29 days of age, the patient developed hypoglycaemia with convulsions, requiring hospitalization for 10 days to treat bronchiolitis. She had normal neuropsychological development. At 1 year of age, her mother noticed that she was shorter than other children of the same age. Her mother`s height is 157 cm and her father’s height is 172 cm, (mid-parental height 158 cm). The patient presented in the clinic at 4.5 years of age with a height of 81cm (−4.9 SDS) and a weight of 10.8 kg (−2.9 SDS). The lack of venous access prevented the performance of a GH provocation test. Her basal IGF1 was undetectable (Table 2), and she presented with a delayed bone age (BA) of 9 months at the chronological age of 3.75 years. Genetic analysis revealed the heterozygous OTX2 p.Q99* mutation in this patient. Ocular ultrasound revealed a completely normal diameter of the eyes for a girl of her age, and she had normal ocular refraction. MRI of the brain revealed a small anterior pituitary with an EPP, a patent and thin pituitary stalk, and normal optic nerves (Table 1). She is currently on rhGH at a dose of 0.033 mg/kg/day and has a growth velocity of 10.6 cm/year. She will be monitored for evolving endocrinopathy. She is, therefore, the first individual in the family who manifests congenital hypopituitarism without an ocular phenotype.

#### Patient 10 (p.H230L)

A male proband was born at term. Short stature was noted at 10 years but he presented in the clinic at the age of 15 years, with no signs of puberty. He was diagnosed with hypopituitarism (GH, TSH, LH/FSH and ACTH deficiencies), polydactyly and normal eyes (Table 1). The patient is now deceased. He had a GH peak of 0.3 µg/L following an insulin tolerance test (Table 2). GH (rhGH 0.033 mg/kg/day), thyroxine and hydrocortisone treatment were started at the age of 15 years, and puberty was induced at the age of 17 years. He had a growth velocity of 11.7 cm per year in the first year following the commencement of GH treatment. MRI of the pituitary gland revealed stalk interruption, APH, and an EPP. Sanger sequencing of *OTX2* revealed a novel heterozygous variant in *OTX2*: c.689A>T, p.H230L. The proband’s mother has short stature (−2.4 SDS) and is a carrier of the p.H230L variant. He had four unaffected siblings (Tables 1 and 2), two of whom are also carriers of the variant and have normal stature (sister with a height −1.7 SDS, brother with a height −0.4 SDS). The mother and siblings have normal basal pituitary hormone concentrations. Following our in-depth studies on this variant, it appears to be tolerated based on analyses in cell culture and mice (see Results).

### Transactivation assays using luciferase reporters

**Figure 3:**
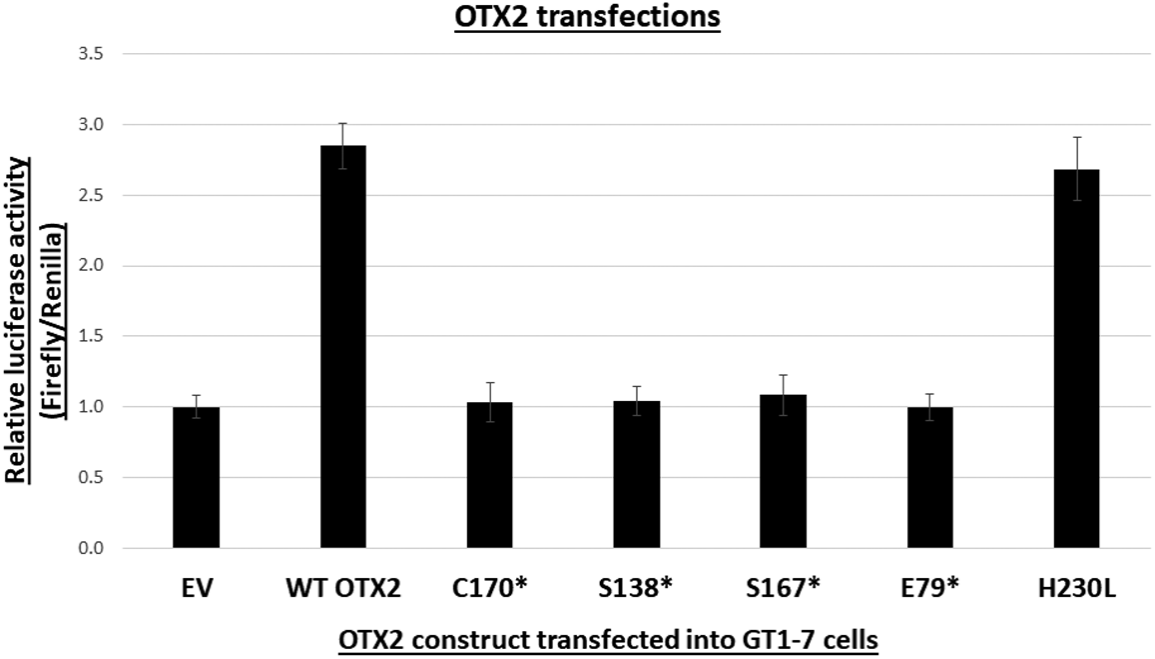
*OTX2* transactivation in GT1-7 cells transfected with mutated *OTX2* constructs. Cells transfected with WT human *OTX2* induced an approximate three-fold increase in transactivation compared to cells transfected with empty vector. Cells transfected with mutated constructs, p.S138*, p.C170*, p.S167*, and p.E79*, respectively, demonstrated a complete loss of transactivation, comparable to cells transfected with empty vector. Contrastingly, cells transfected with the p.H230L construct had comparable transactivation properties to WT OTX2, yielding no significant difference. Values are normalized to Renilla luciferase, and then normalized to the value taken from cells transfected with an empty vector. These results were generated from three independent experiments in triplicate. Error bars represent +/− standard deviation.

Cells transfected with humanwt*OTX2* and a luciferase reporter bearing multiple OTX2 consensus binding sites induced a three-fold increase in luciferase activity relative to the empty vector (Fig. 3). A complete loss of OTX2 activity was observed for cells transfected with *OTX2* expression vectors with the mutations p.S138*, p.C170*, p.S167*, and p.E79* (Fig. 3). In contrast, the p.H230L variant was not significantly different from wt OTX2.

### Otx2^H230L^ variant mice were normal, but Otx2^L219fs^ mice had eye abnormalities

**Figure 4:**
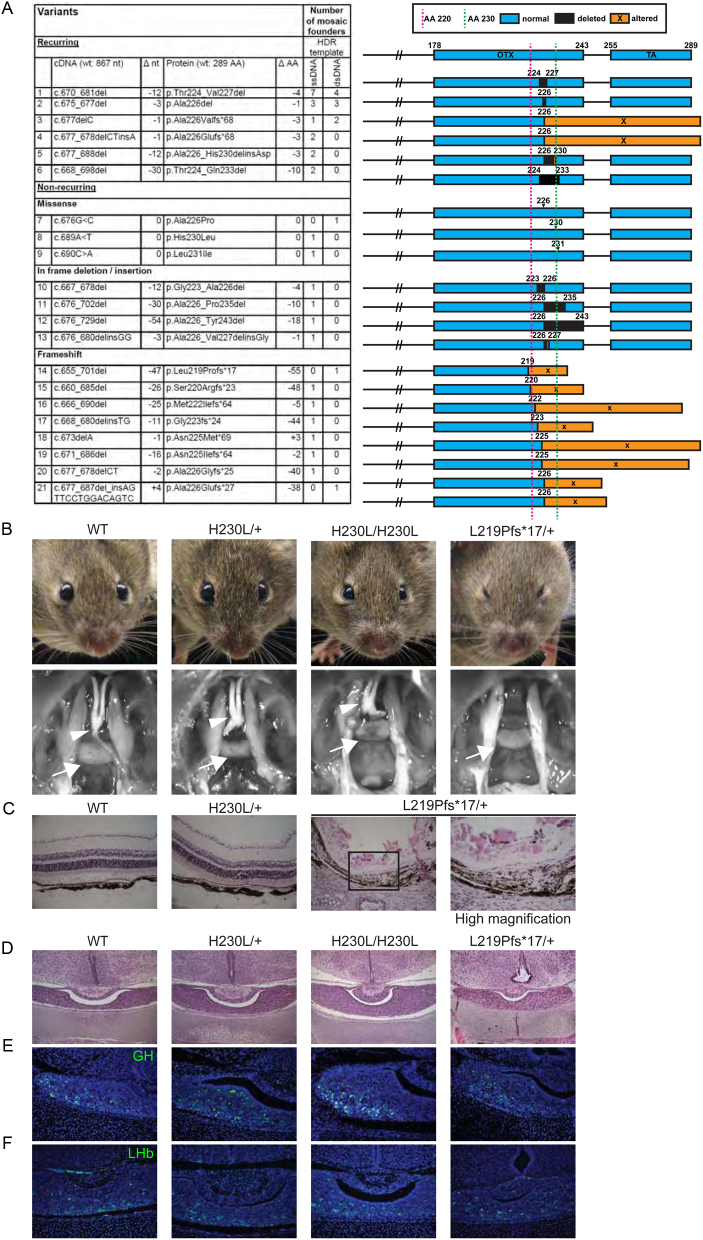
OTX2^H230L^ had no abnormal phenotype, and C-terminal variants are tolerated. (A) CRISPR/Cas9 generated *Otx2*^H230L^ and multiple by-product variants. (B) *Otx2*^H230L^ had no obvious phenotype in either the pituitary gland or the eye. *Otx2*^L219fs*17^ showed anophthalmia/microphthalmia but, however, showed similar pituitary morphology to WT animals. Arrows and arrowheads indicate the pituitary gland and optic nerve, respectively. (C) HE staining of eyes of adult mice. *Otx2*^H230L^ had normal eyes, but the retina of *Otx2*^L219fs*17^ was thin. (D, E and F) Pituitary histology of P0 mice. (D) HE staining, (E) GH staining, and (F) LH staining were similar between genotypes.

The *Otx2*^H230L^ variant retained transactivation properties in transfected cells (Fig. 3). However, the amino acid change is located within the conserved OTX domain which is involved in protein–protein interactions (11) and may, therefore, not affect transactivation in transient transfection assays. Thus, to clarify the effect of this variant on pituitary development, we generated *Otx2^H230L^* mice using the CRISPR/Cas9 system (Fig. 4A). We also created multiple variants ofthe *Otx2* gene as by-products of the p.H230L variant. These included frameshifts and deletions around the site of the guide RNA. The majority of the variants, including *Otx2*^H230L/+^ mice, did not show any external deformity (Fig. 4A). The variant that is predicted to cause the largest C-terminal truncation, *Otx2*^L219fs*17/+^, elicited eye abnormalities with complete penetrance. The abnormalities included either unilateral or bilateral anophthalmia or microphthalmia. Gross autopsy findings revealed that *Otx2*^H230L^ heterozygotes and homozygotes had normal pituitary glands and optic nerves (Fig. 4B). The *Otx2*^L219fs*17/+^ mice lacked optic nerves, however, the pituitary gland appeared normal, and the mice were of normal size. Histological analysis of *Otx2*^H230L/+^ mice revealed a normal organization of the retinal layers (Fig. 4C). In contrast, the eye histology of *Otx2^L^*^219fs*17/+^ was obviously abnormal. The retina was thin, the cells of both outer and inner nuclear layers were sparse, and the retinal pigmented epithelium was disorganized. As OTX2 is important for the production of gonadotropin-releasing hormone (GnRH) (27), and GH is the most affected hormone in patients with CH, we analysed the expression of GH and LH in the pituitary gland using immunostaining. The expression of these hormones appeared normal in both *Otx2*^H230L/+^ and *Otx2*^L219fs*17/+^ mice (Fig. 4D, E and F). Taken together, this allelic series of *Otx2* variants suggest that truncation of the OTX2 C-terminal to amino acid 220 does not impact on eye development and that the p.H230L variant is tolerated.

### In situ hybridisation on human embryonic sections

**Figure 5:**
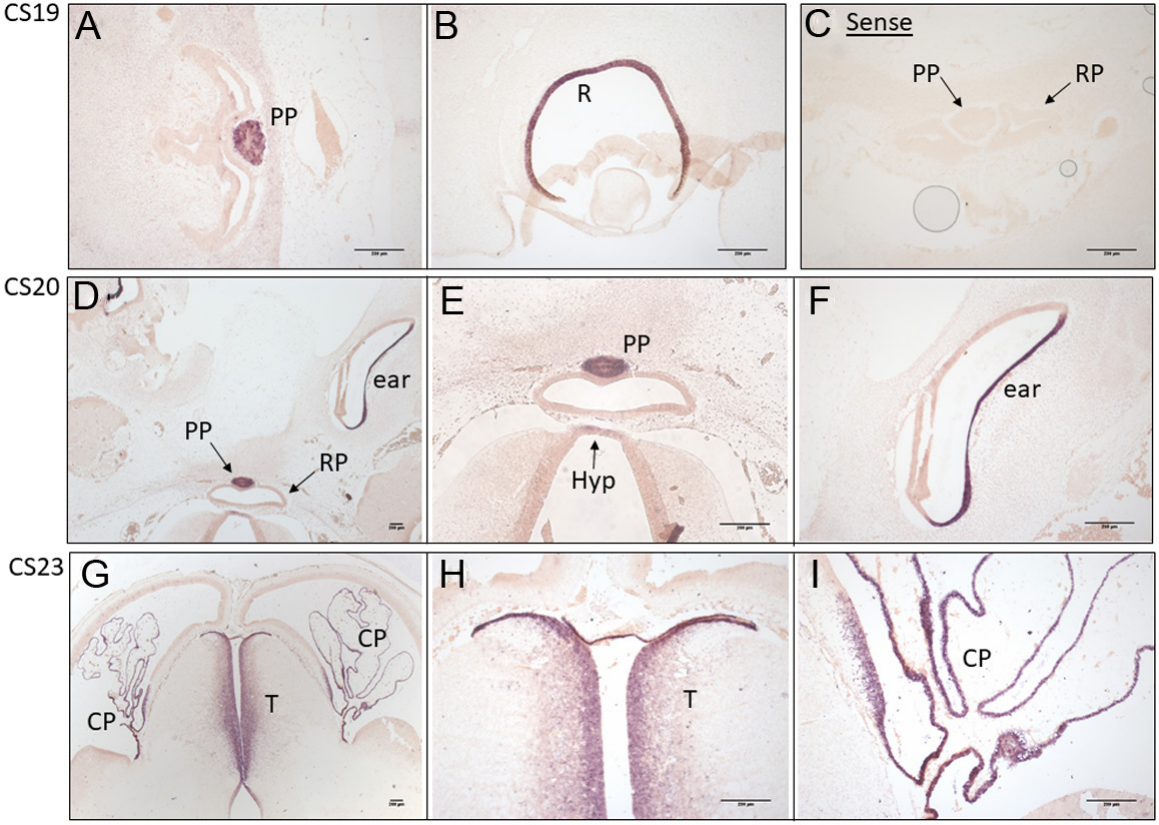
Human *OTX2* expression at different stages during embryogenesis. (A and B) Carnegie stage (CS) 19. (A) Strong human *OTX2* mRNA transcript expression in the posterior pituitary and (B) in the retina of the eye. (C) No expression was observed in Rathke’s pouch or the posterior pituitary using the control sense probe. (D) CS20. *OTX2* expression is maintained in the posterior pituitary and is seen in the developing ear. (E and F) Magnified images of ‘D’ showing partial expression in the hypothalamus (indicated by the labelled arrow), and strong expression in the inner ear. (G) CS23. Strong transcript staining in the thalamus and choroid plexus. (H and I) Magnified images of ‘G’ showing the gradient of *OTX2* expression in the thalamus and the strong expression in the choroid plexus. PP, posterior pituitary; R, retina; RP, Rathke’s pouch; Hyp, hypothalamus; CP, choroid plexus; T, thalamus.

Human *OTX2* mRNA transcripts are expressed exclusively in the posterior lobe of the pituitary at CS19 and 20 (6 and 7 weeks GA) during embryogenesis, however, not in Rathke’s pouch (Fig. 5A, D and E). *OTX2* expression is also detected in the developing retina of the eye and the ear at CS19 and CS20 (Fig. 5B, D and F). There is some partial staining in the hypothalamus in the tip nearest to Rathke’s pouch, at CS20 (Fig. 5D and E). There was no staining at CS16 (5.5 GA) in any tissue analysed. *OTX2* transcripts are strongly expressed in the human thalamus and choroid plexus at CS23 (8 weeks GA) (Fig. 5G, H and I), however, with no staining in the pituitary or hypothalamus at this stage.

## Discussion

OTX2 regulates the development of the anterior pituitary through the activation of *HESX1* and *FGF8* expression and has an essential role in the anterior structure and forebrain maintenance (6, 28). We identified 7/127 loss-of-function variants in UK patients with SOD and accompanying eye defects (5.5%), similar to the reported 3% published in the literature (14). Haploinsufficiency of *OTX2,* due to heterozygous absence of the entire gene, within both identified chromosomal deletions del(14)(q22.2–23.31) and del(14)(q22.1q23.1), was predicted to be the most likely pathogenic cause for the CH and eye defects described in patients 1 and 2. However, we cannot completely rule out the contribution of other deleted genes within this region towards the phenotype in both patients. The *de novo* heterozygous p.S138* mutation, present in patient 3, was first reported in a patient with early onset retinal dystrophy, GHD, mild torsional nystagmus and alternating esotropia, with a normal MRI (4). Patient 3 has the same phenotype of retinal dystrophy and GHD but with a small anterior pituitary, a small infundibulum, and an EPP on their MRI. This variant was predicted to lead to a loss in transactivation function (4) based on previous studies of *OTX2* truncations at residues 106 and 161 (29). Our study supported this hypothesis through functional assays showing a significant decrease in transactivation activity compared to WT. This is similar to the other truncations studied, namely p.C170*, p.S167* and p.E79*. The p.C170* mutation in patient 4 has not been described in the literature; however, it was identified in a genome sequencing project performed by Eurofins (www.eurofinsgenomics.eu) in a patient with bilateral complete anophthalmia and congenital heart disease, further details of which are unavailable. The p.E79* variant in patient 5, where a missense substitution to lysine has been described before at the same position (p.E79K) in a pedigree with pattern dystrophy of the retinal pigment epithelium (30), the p.S167* and the p.Val139Asp*39 variants in patients 6 and 7 respectively, were novel. The p.Val139Asp*39 and p.Q99* variants respectively, were predicted to yield a similar loss in transactivation function to the other frameshifts and were not functionally studied.

Additionally, we screened a cohort of 282 Brazilian patients with IGHD or CPHD, with 18/282 having additional eye abnormalities. The p.Q99* mutation identified in patient 8, that was previously described as part of a targeted panel result (22), was subsequently identified in his affected niece, patient 9. Six other individuals within this pedigree also carried the variant and exhibited a range of phenotypes including normal, microphthalmia, GHD, and an ear malformation. Interestingly, patient 9 does not have an ocular phenotype, in contrast to her maternal uncle (patient 8). The range of phenotypes present within this pedigree is consistent with incomplete penetrance and variable expressivity noted in other familial *OTX2* cases (13, 15). Furthermore, in contrast to the phenotype seen in most patients with OTX2 mutations, this family presents a rare example of OTX2 haploinsufficiency manifesting as a hypopituitarism phenotype in the absence of accompanying eye defects (patient 9, IV.2 in Fig. 2A). This mutation was previously reported in a male (inherited from his unaffected father) with bilateral anophthalmia, hippocampal malformation, seizures, and severe developmental delay (13); however, there was no information regarding his pituitary function.

The single missense variant identified in the Brazilian CH cohort, p.H230L (patient 10), showed no significant difference in transactivation assays compared to wt *OTX2* and was tolerated in transgenic mice. Thus, this variant is most likely not causative of hypopituitarism. Other variants are currently under investigation in this family.

OTX2 has three evolutionarily conserved protein functional domains: the DNA binding homeodomain (HD; amino acid (AA) 38–97), the OTX domain (AA 148–245), and the transactivation domain (AA 255–289) (11, 21). The OTX domain has been found to be involved in protein interactions with LHX1, while the HD and its C-terminal side bind to FOXA2 (HNF-3β) or OTX2 itself (31) OTX2 interaction with LHX1 enhances transcription, while interaction with FOXA2 suppresses it. Interestingly, mice heterozygous for truncations C-terminal to aa220 (within the OTX domain) had normal eye development, while truncation at aa219 and earlier was not tolerated. This suggests that the most critical functional domains of OTX2 lie in the first 220 amino acids. However, we cannot rule out the effect of modifier genes segregating in the cross.

We detected strong *OTX2* expression in the posterior pituitary, developing ear, and retina at CS19 and 20 (6–7 weeks GA), and in the thalamus and choroid plexus at CS23 (8 weeks GA), during human embryogenesis, supporting previous human expression work (32). Expression was also observed in the hypothalamus tip nearest to Rathke’s pouch at CS20. This pattern of gene expression is consistent with the range of tissues affected in patients heterozygous for *OTX2* loss-of-function alleles. There are no detectable *OTX2* transcripts in Rathke’s pouch from as early as CS16 (5.5 weeks GA) in our study. In mice, OTX2 immunostaining was robust in the ventral diencephalon and prospective posterior lobe from E10.5–E14.5 (3, 16), which is the approximate equivalent of CS12–19 in humans; however, no *Otx2* transcripts were detected in Rathke’s pouch from E11.5–E14.5 (Allen Developing Mouse Brain Atlas (2008)). Furthermore, conditional disruption of *Otx2* in the developing diencephalon led to reduced *FGF8* expression and poor anterior pituitary growth (3). Together, these data support the idea that anterior pituitary dysfunction is secondary to defects in the developing brain and posterior pituitary development (3, 20). Previous reports of human foetal *OTX2*/OTX2 expression identified both mRNA transcripts and protein in the diencephalon and mesencephalon at 7 foetal weeks (fw), in the basal telencephalon, hippocampus and the choroid plexus at 7 and 9 fw, and in the thalamus at 9 fw, similar to our findings. OTX2 is secreted by the choroid plexus and transferred to supporting cells of the ventricular-subventricular zone, which is responsible for generating olfactory bulb neurons and the rostral migratory stream in mice. Conditional knockout of *Otx2* in the choroid plexus affects neuroblast migration and reduces the number of olfactory bulb neurons in newborns (33). The choroid plexus is likely the most prominent source of OTX2 release, which stimulates the generation of sensory neurons elsewhere in the brain during development. We detected *OTX2* transcripts in the developing ear and retina, in keeping with reports of expression in the retinal pigment epithelium (34). These data may explain the association of *OTX2* mutations with sensory problems such as deafness and ear abnormalities (as observed in patients 1, 7, and 8), and possibly hypoplastic olfactory bulbs, as well as the more commonly described eye abnormalities.

All of the patients we report with *OTX2* mutations in our large multi-ethnic cohort had hypopituitarism and variable eye abnormalities. These patients had structural defects of the pituitary gland, such as a hypoplastic anterior pituitary and an EPP, similar to other patients with *OTX2* mutations. No *OTX2* mutations were found in 264 Brazilian patients with hypopituitarism without eye abnormalities, with the exception of patient 9, consistent with previous studies such as that reported by Dateki * et al.*, where no *OTX2* mutations were identified in 66 patients with hypopituitarism and no ocular abnormalities, as opposed to 5/28 being described in patients with ocular malformations (35). Like many other patients with congenital hypopituitarism, GHD is the most common endocrinopathy in patients with an *OTX2* mutation, as seen in patients 1-10. The majority of patients had classical GHD with a low GH peak; however, two of these patients had a normal GH but a low IGF1 and a low/normal IGFBP3 (patients 3 and 7). It is important to note that false-negative results with a GH peak greater than 10 µg/L have been reported for the glucagon test in children with congenital hypopituitarism and EPP (36), and this is likely to be the case with patient 3. Patient 7 will require careful monitoring, as it is highly likely that she will develop GH deficiency with/without other pituitary hormone deficiencies. Patient 4 did not have a provocation test performed as he was too young, however, he was failing in terms of growth and had a low IGF1. Other pituitary hormone deficiencies have evolved in previous cases, such as the central hypothyroidism seen in Patient 2; therefore, patients need to be closely monitored for evolving endocrinopathy (37).

This study highlights the incomplete penetrance and variable phenotypes that may manifest in patients within the same family with OTX2 haploinsufficiency (p.Q99*) (Fig. 2A). This pedigree demonstrates variability in the penetrance of OTX2 through the p.Q99* mutation. The severe phenotype of microphthalmia and GHD in patient 8 and the milder phenotype of GHD without ocular malformations in patient 9 are in direct contrast to the three asymptomatic carriers; the mother (IV.2), grandmother (II.5) and great-grandmother (I.2) of patient 9 who carry the same mutation. Other family members have serious yet isolated features: a cardiac malformation (II.2), an ear malformation (III.3) and microphthalmia (III.5) without GHD, possibly highlighting the highly variable phenotypes stemming from haploinsufficient OTX2. We describe the third case to date, of a patient with an *OTX2* mutation with congenital hypopituitarism without eye abnormalities (patient 9). This is the first OTX2 frameshift/early stop codon mutation, as opposed to two previous *OTX2* missense variants, reported in a patient with hypopituitarism in isolation. This phenotype differs from the severe eye defects that usually accompany hypopituitarism in the majority of patients with *OTX2* mutations.

To conclude, human *OTX2* expression in the posterior pituitary is consistent with a critical role for OTX2 in the development of the neural ectoderm that gives rise to the hypothalamus, pituitary stalk, and posterior lobe. Itis necessary for the robust expression of signalling molecules that stimulate the growth of Rathke’s pouch, such as bone morphogenetic protein 4 (BMP4) and fibroblast growth factors (FGFs) 8 and 10 (3). Hypothalamic-specific *Otx2* knockout mice exhibit weak expression of these molecules, suggesting that reduced anterior pituitary gland growth during development is secondary to OTX2-dependent activation of BMP and FGF signalling (3). The absence of *OTX2* transcripts in Rathke’s pouch is consistent with this mechanism. We identified two chromosomal deletions spanning *OTX2*, and six pathogenic *OTX2* variants (three of which are novel), in IGHD and CPHD patients with and without eye malformations. From these 9 patients with intolerant *OTX2* variants (Table 1), 6/9 had developmental delay, 6/9 had IGHD, and 7/9 had an eye phenotype ranging from retinal dystrophy or ONH to the more severe microphthalmia. We cannot exclude that the five surviving patients may develop additional anterior pituitary defects over time based on the high probability of risk that characterizes patients presenting with EPP on MRI examination. In addition, our data suggest that the majority of patients with *OTX2* mutations have developmental delays.

## Supplementary Material

Supplementary Figure 1

## Declaration of interest

The authors declare that there is no conflict of interest that could be perceived as prejudicing the impartiality of this study

## Funding

NIH grant R01 HD30428 (SAC) and the Transgenic Animal Model Core facility funding NIH NIDDK34933. Brazilian patient studies thank the NIH WRHR Scholar grant 1K12HD065257 and Nastaran Foyouzi for her contributions to the early stages of this work. Fundação Faculdade de Medicina and FAPESP (Fundação de Amparo à Pesquisa do Estado de São Paulo – LRC, AALJ, IJPA, BBM). The Japan Society for the Promotion of Science http://dx.doi.org/10.13039/501100001691 Overseas Research Fellowship (H B). M T D receives funding from the Great Ormond Street Hospital (GOSH) Children’s Charity and the Medical Research Foundation http://dx.doi.org/10.13039/501100009187, UK (grant# 535963). Research at GOSH benefits from funding received from the NIHR Biomedical Research Centre (L C G, M T D). The human embryonic and foetal material were provided by the Joint MRC/Wellcome Trust http://dx.doi.org/10.13039/100010269 (Grant MR/R006237/1) Human Developmental Biology Resource (HDBR) (http://hdbr.org).

## Author contribution statement

S A Camper, L R S Carvalho and M T Dattani contributed equally to this work.
