# Supplementary material for: The phenotypic spectrum associated with OTX2 mutations in humans
Source: Eur J Endocrinol. 2021 May 5;185(1):121–35. doi: 10.1530/EJE-20-1453 (PMC8437083; doi:10.1530/EJE-20-1453)
Supplement: Supplementary Figure 1 [file supplementary_figure_1.pdf]

Supplementary Figure 1

A

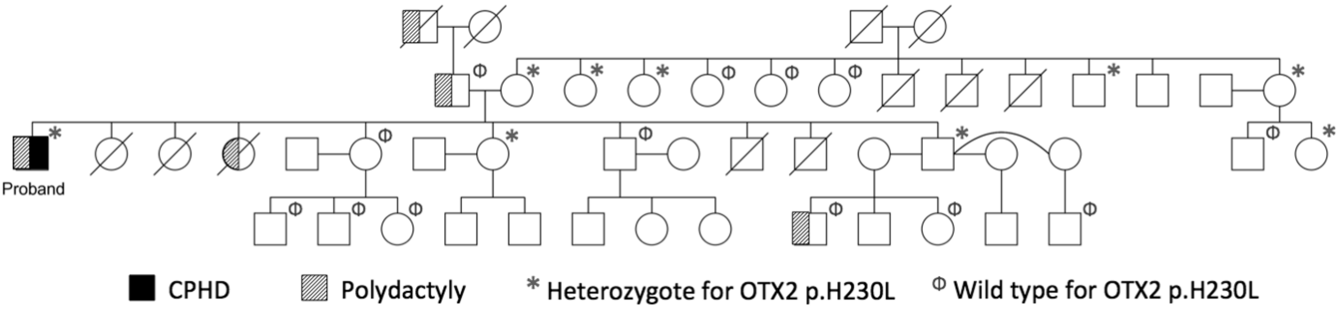

B

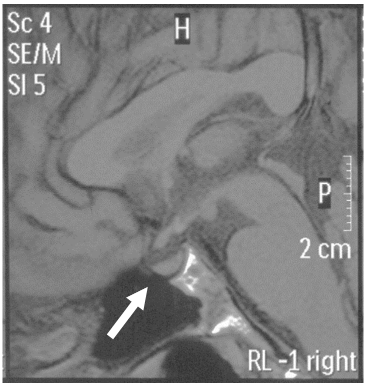

C

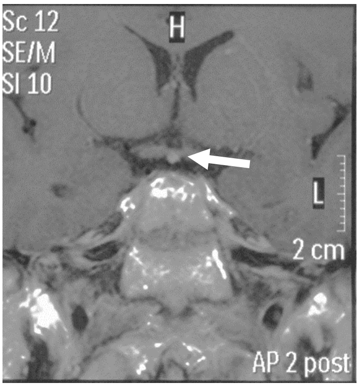

D

Gene	Transcript	Variant	cDNA	Allele	Protein	Variant effect prediction				MAF					SNP ID
						CADD	SIFT	Polyphen2	MutationTaster	ABraOM	gnomAD	ExAc	1000G	ESP	
OTX2	ENST00000408990	14:57268634 T / A	c.689A>T	Heterozygous	p.H230L	24.5	deleterious	possibly damaging	disease causing	None	8.0E-06	8.2E-06	None	7.7E-03	rs144449264

E

```
Conservation   cDNA               Protein

Human          ACCAGCCATCTCAAT  | N A V T S H L N Q S P
Mouse          ...............  | . . . . . . . . . . .
Sheep          ...............  | . . . . . . . . . . .
Chicken        ........C.....C  | . . . . . . . . H . .
Frog           ..A..T..C..T..C  | . . . . . . . . . . .
Zebrafish      .....T..C..A...  | . . . . . . . . . . .
```
